# Research hotspots and trends in visceral pain research: A global comprehensive bibliometric analysis

**DOI:** 10.3389/fnmol.2022.1022463

**Published:** 2023-01-04

**Authors:** Le Guan, Yang Liu, Bin Wu, Aiqin Chen, Wucheng Tao, Chun Lin

**Affiliations:** ^1^Key Laboratory of Brain Aging and Neurodegenerative Diseases, School of Basic Medical Sciences, Pain Research Institute, Fujian Medical University, Fuzhou, Fujian, China; ^2^Key Laboratory of Brain Aging and Neurodegenerative Diseases, School of Basic Medical Sciences, Fujian Medical University, Fuzhou, Fujian, China; ^3^Department of Gastroenterology, First Affiliated Hospital of Nanchang University, Nanchang, Jiangxi, China

**Keywords:** anxiety, inflammation, quality of life, bibliometric analysis, visceral pain

## Abstract

**Background:**

Visceral pain is a complex and heterogeneous disorder that is considered more prominent compared to somatic pain, due to its multiple and complex causes and accompanying emotional and mood disorders. Research has become increasingly extensive over the years, but a bibliometric analysis of this field is lacking. The aim of this study was to analyze global research trends in visceral pain over the past 40 years through visual analysis.

**Methods:**

We conducted a comprehensive search of the literature from January 1981 to December 2021 using the Web of Science core database. The medical subject term ‘visceral pain’ was searched. We used CiteSpace and VOSviewer for bibliometric analysis and network visualization, including top-ranked authors, keywords, research collaborations, and literature co-occurrence network analysis.

**Results:**

A total of 5,047 articles were included in the analysis. The number of articles on visceral pain has continued to grow steadily over the past 40 years. The United States (1,716 articles), University of California (159 articles), and Neurogastroenterology and Motility (276 articles) were the country, institution, and journal with the most publications, respectively. Keyword analysis showed that inflammation, visceral hypersensitivity, inflammatory bowel disease (IBD), irritable bowel syndrome (IBS), anxiety, and quality of life were the research trends and priorities in this research field.

**Conclusion:**

Visceral pain-related research has received increasing attention in recent decades. However, there are still many unresolved issues in the field of visceral pain, such as the specific molecular mechanisms and clinical treatments between visceral pain and inflammation, IBD, IBS, anxiety, and quality of life, which may require further exploration based on modern scientific and technological means and more basic research, especially for the therapeutic targets of visceral pain, which may become a hot spot for future research and provide guidance for the treatment of clinical diseases related to visceral pain.

## 1. Introduction

Visceral pain is a complex and heterogeneous disease with modulation involving multiple regions including the brain and spinal cord. It includes common acute (digestive/intestinal ulcers, cholecystitis, etc.) and chronic symptoms (functional abdominal pain, intrauterine membrane endometriosis, chronic pain of the gut, etc.; Grundy et al., 2019). Some of these pains have clear underlying pathologies that can be treated for the cause. But in many cases the pain has no pathology, which is called idiopathic or functional disorder. This is probably associated with stress and mood changes (Coen et al., 2009; Rosenberger et al., 2009). Because the mechanisms that cause or sustain functional disorder are not fully understood, the treatment strategy has not been found. It has been shown that manipulating emotions through fear images, music, cognitive or emotional stress alters subjective perceived pain ratings during visceral stimulation (Gebhart and Bielefeldt, 2016). Therefore, the strategy of treating functional abdominal pain should be considered in this regard. Currently, many researchers are investigating visceral pain because of the increased disease burden of this type of pain. More than 20% of the world’s population suffers from chronic visceral pain (Grundy et al., 2019; Louwies et al., 2019). One third of the US population reported pain that had lasted for more than 6 months, and chronic pain and discomfort negatively impact quality of life for those affected (Johannes et al., 2010; Gebhart and Bielefeldt, 2016; Pusceddu and Gareau, 2018; Shaballout et al., 2021; Ceuleers et al., 2022; Jain et al., 2022; Long et al., 2022; Morreale et al., 2022; Zhang et al., 2022).

However, no systematic analysis of this area has been carried out using bibliometric methods. Bibliometric analysis was introduced by Pritchard in 1969 and plays an important role in reflecting characteristics and future trends (Tan et al., 2021; Zou and Sun, 2021). It is a field of library and intelligence research involving the quantitative analysis of literature, which can estimate the development trends of a certain field and reveal the key research directions by analyzing database and literature characteristics, helping scholars to quickly understand research hotspots and development trends (Ma et al., 2020, 2021; Wang et al., 2021; Chen et al., 2022).

The aim of this study was to analyze the current research status and development trends related to visceral pain published between 1981 and 2021 through a comprehensive analysis of bibliometric methods. More specifically, the study aimed to analyze the relevant research trends and hot spots in the field of visceral pain from the perspective of countries, journals, authors, keywords, and highly cited literature, which will be of great importance for scholars in this field to enable them to determine future research directions (Zhou et al., 2021).

## 2. Materials and methods

### 2.1. Data sources and search strategy

The Web of Science (WOS) Core Collection database is a commonly used database in bibliometric analyses (Zhang et al., 2021). It can provide complete information on bibliometric requirements and is the most influential database of its type. We therefore conducted a comprehensive search of the literature from January 1981 to December 2021 using the WOS core database. The medical subject term ‘visceral pain’ was searched.

### 2.2. Inclusion and exclusion criteria

**Figure 1:**
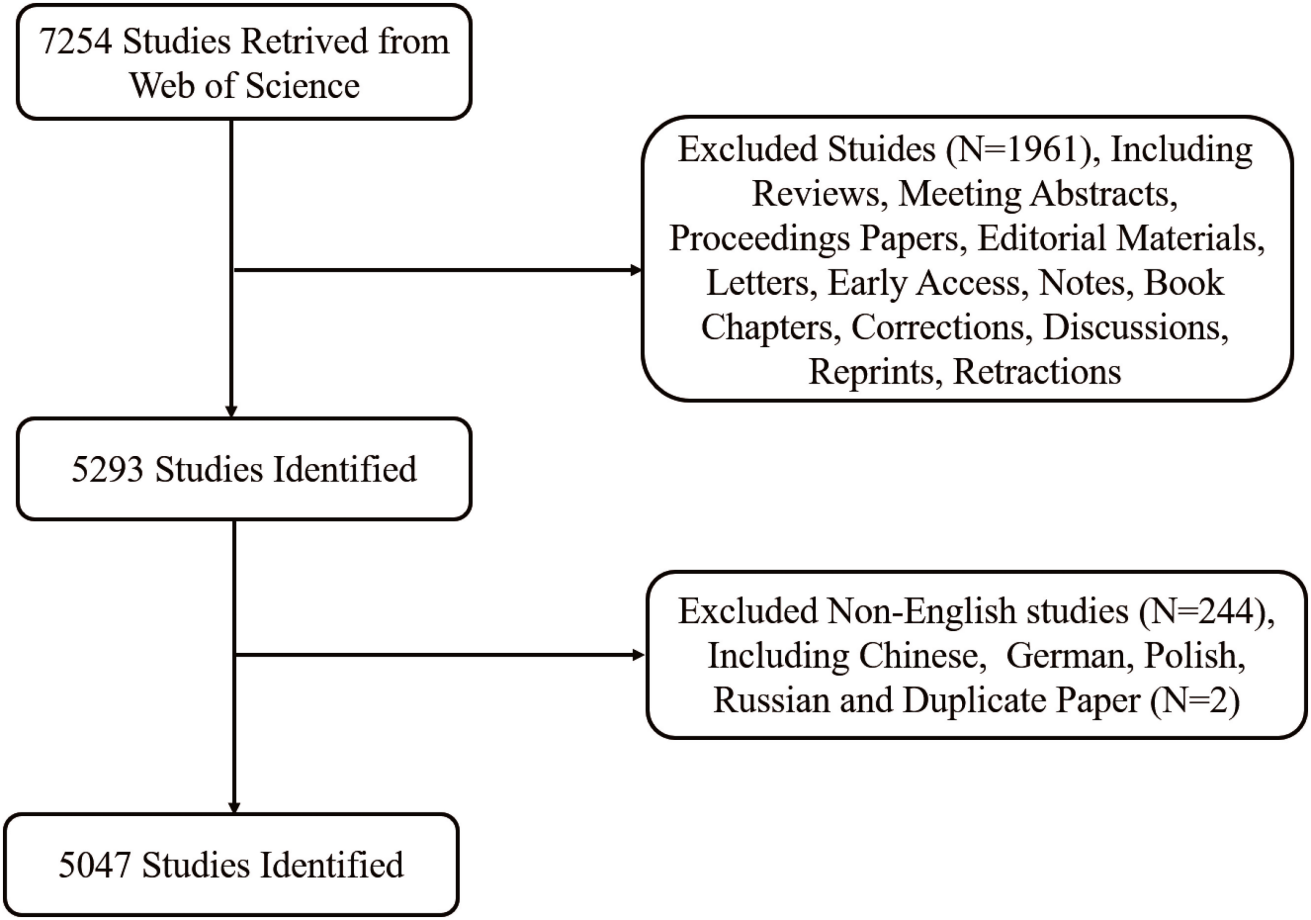
Flowchart of literature selection.

The data used in this study were downloaded from the WOS core database, and a total of 7,254 relevant articles were retrieved after careful screening of titles, abstracts, and author keywords by two independent authors (GL and LY). We restricted included article types to articles, and the language of publication of articles was limited to English. The excluded literature comprised of 1,961 articles other than articles (Reviews, Meeting Abstracts, Proceeding Papers, Editorial Materials, Letters, Books, Revisions, and Reprints), 244 non-English studies (Chinese, German, Polish, and Russian), resulting in 5,049 remaining articles. These data were downloaded and imported into CiteSpace software to clean and remove duplicates and remove two articles, ultimately identifying 5,047 articles for analysis. The detailed screening process is shown in Figure 1. The flow chart of the entire bibliometric analysis is detailed in Supplementary Figure S3.

### 2.3. Analytic methods

We used CiteSpace and VOSviewer for bibliometric analysis and network visualization, including top-ranked authors, keywords, research collaborations, and literature co-occurrence network analysis (Chen et al., 2020). CiteSpace is a visualization software for bibliometric analysis developed by Professor Chaomei Chen (Drexel University, USA) (Zhu et al., 2020; Li et al., 2022). It is an interactive analysis tool that enables visualization tasks in scientific mapping by combining bibliometrics, visual analysis methods, and data mining algorithms, focusing on: analyzing and predicting trends and hot spots in various fields; visualizing relationships between countries, regions, organizations, and authors; and summarizing authors, journals, and references (Zhu et al., 2020; Zhou et al., 2021). VOSviewer is a program used to build and view bibliometric maps for constructing authors or journal co-citations, authors, references, and their citation data based on collaborative data that, together with the visual maps created by CiteSpace, reflects collaborative and scholarly relationships (Ma et al., 2021; Tan et al., 2021; Zhou et al., 2021). We used CiteSpace 6.1.R2 and VOSviewer version 1.6.16 (Chen et al., 2022) to analyze the data acquired by WOS for collaborative relationships, co-citations, and keyword co-occurrence (Zhou et al., 2021). We conducted a literature search and downloaded the raw data on the date of July 14, 2022, to reduce bias due to frequent database updates. The raw data for inclusion in the study were initially downloaded from the WOS core database as plain text files, from which we extracted information such as title, author, institution, country, year of publication, references, and keywords, and saved them in TXT format. The color of the elements in the figure represents the clusters to which they belong, and different clusters are represented by different colors. The view allows one to view each individual cluster, for example, to discover the structural distribution of research hotspots by topic co-occurrence, research groups by author collaboration, and the similarities and differences among scholars on research topics by author coupling networks. In addition, we used GraphPad Prism 9.0.0 and Microsoft Office 2019 software to create charts.

Journal Citation Reports (JCR) is an important reference indicator commonly used to evaluate the quality and level of journals. The journals are divided into 176 different subject categories, and each subject category is divided into four zones according to the impact factor of the journals: the top 25% impact factor journals comprise Zone Q1; the top 25%–50% impact factor journals comprise Zone Q2; the top 50%–75% impact factor journals comprise Zone Q3; and the remaining journals comprise Zone Q4.

## 3. Results

### 3.1. Annual growth trends

**Figure 2:**
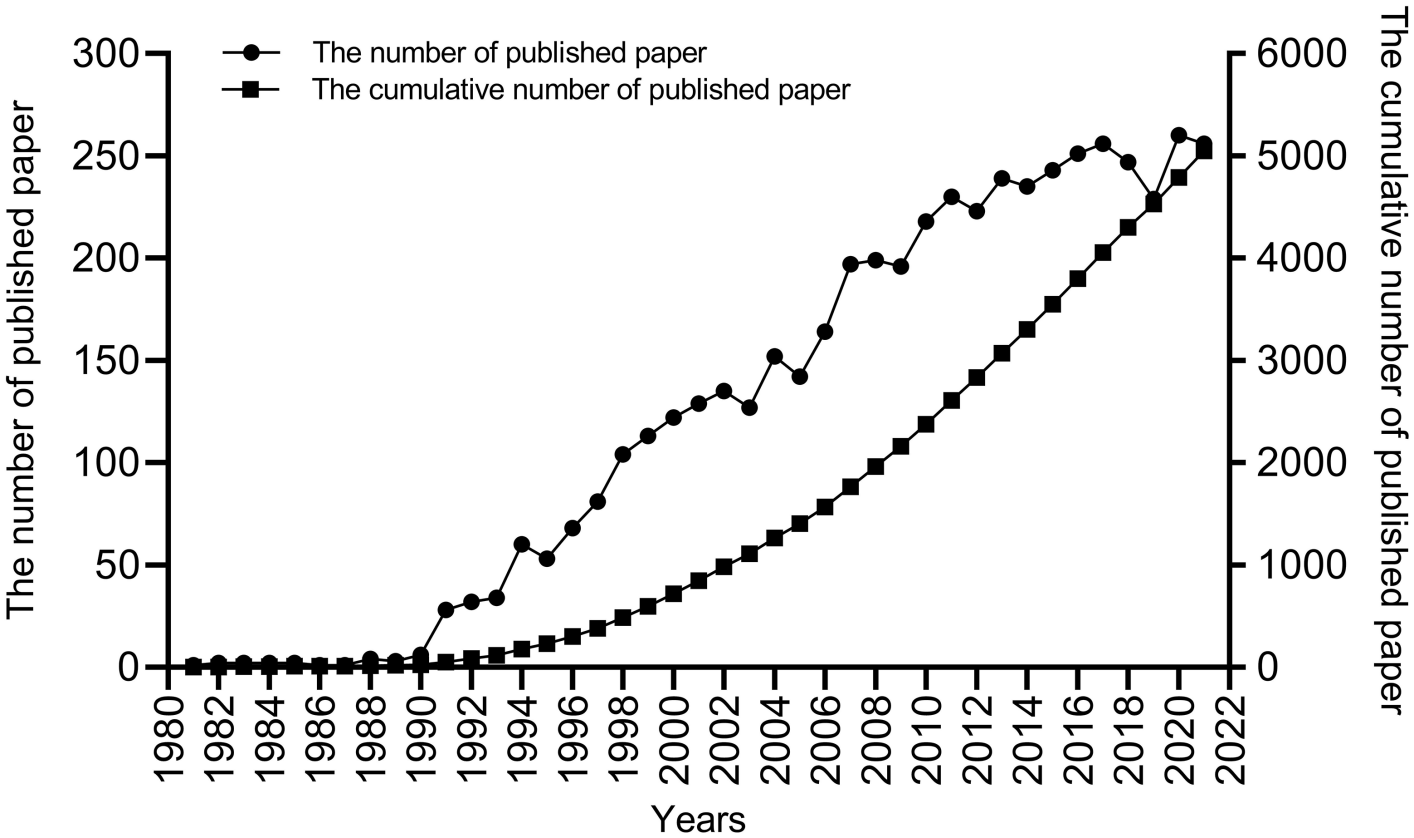
The number and cumulative number of publications in the period from 1981 to 2021.

The change in the number of papers published annually is an important indicator, and the number of articles published in each period provides a visual indication of the research trends in the field. According to our search strategy, a total of 5,047 publications related to visceral pain were published between 1981 and 2021. It can be seen from Figure 2 that relevant publications have grown rapidly since 1991. Although the number of publications showed a decreasing trend in 1995, 2003, 2005, 2009, 2012, and 2019, the cumulative number of publications per year showed a rapid and steady increasing trend. Based on the growth curve of the number of published related papers, we conclude that visceral pain is receiving increasing attention in the field of neuroscience worldwide; it is gradually becoming a major research topic and even a future research hotspot and direction.

### 3.2. Analysis of authors and co-authorship

**Figure 3:**
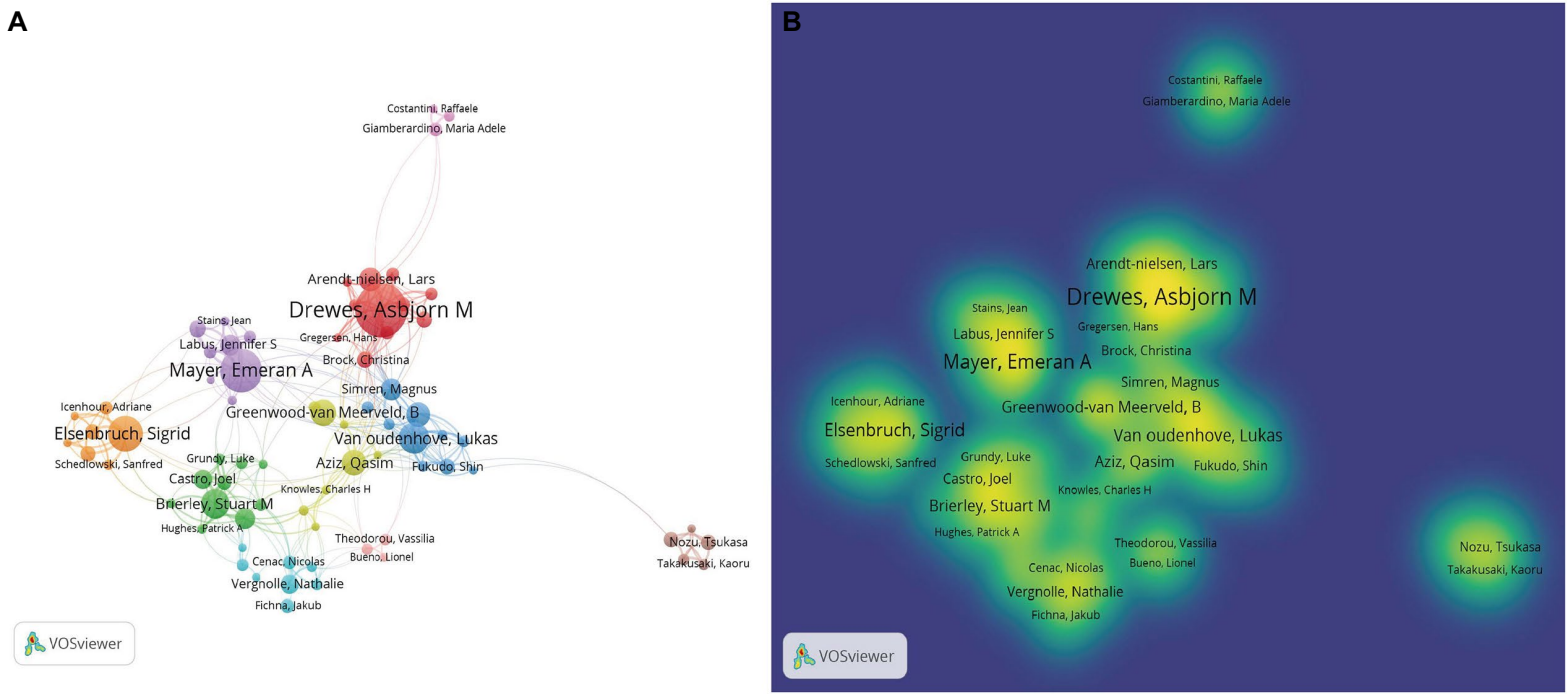
**(A)** The Co-authorship map of Authors in visceral pain research (*T* ≥ 10). The size of node reflects the author’s co-authored papers, the link indicates the co-authored relationship between authors, and the same color of node represent the same cluster. **(B)** The density map of authors in Visceral Pain Research (*T* ≥ 10). Notes: The size of word, the size of round, and the opacity of yellow is positively related to the co-authored papers.

There were 20,905 authors associated with the 5,047 identified articles, and as shown in Table 1, the top 10 authors published a total of 407 articles (accounting for 8.07%). The highest number of articles was by Asbjørn Mohr Drewes (61 articles, accounting for 1.21%), followed by Gerald F. Gebhart (59 articles, accounting for 1.17%), Emeran A. Mayer (49 articles, accounting for 0.97%), Sigrid Elsenbruch (40 articles, accounting for 0.79%), and Guang-Yin Xu (38 articles, accounting for 0.75%). In terms of author citations, the top 5 most cited authors were Gerald F. Gebhart (Citations: 4,283), Emeran A. Mayer (Citations: 3,150), Asbjørn Mohr Drewes (Citations:1,942), Lilian L Bueno (Citations:1,748), and Stuart M Brierley (Citations:1,737; Table 1). Authors with more than 10 publications in the field of visceral pain research were selected, optimized, and mapped for collaboration among 73 authors (Figure 3A), with node size reflecting the number of co-authored papers, connecting lines indicating the existence of collaborative relationships between authors, and the same color indicating the same cluster (10 clusters), suggesting groups of authors in similar research areas. Subsequently, the density map between authors of visceral pain-related research was further plotted by collaborative mapping (Figure 3B). The density map was produced with the same parameters as those set by collaborative mapping, and the size of the font, the size of the circle, and the opacity of the yellow color in the density map were positively correlated with the number of co-authored papers.

**Table 1:** The top 10 productive authors.

Rank	Author	Country	*N*	Citations
1	Asbjørn Mohr Drewes	Denmark	61	1942
2	Gerald F. Gebhart	United States	59	4,283
3	Emeran A. Mayer	United States	49	3,150
4	Sigrid Elsenbruch	Germany	40	1,218
5	Guang-Yin Xu	China	38	814
6	Lukas Van Oudenhove	Belgium	35	1,196
7	Stuart M Brierley	Australia	33	1737
8	Lilian L Bueno	Brazil	33	1748
9	Beverley Greenwood-VanMeerveld	United States	30	825
10	Qasim Aziz	United Kingdom	29	1,451

*N*: The number of publications.

### 3.3. Analysis of the institutions and countries

**Figure 4:**
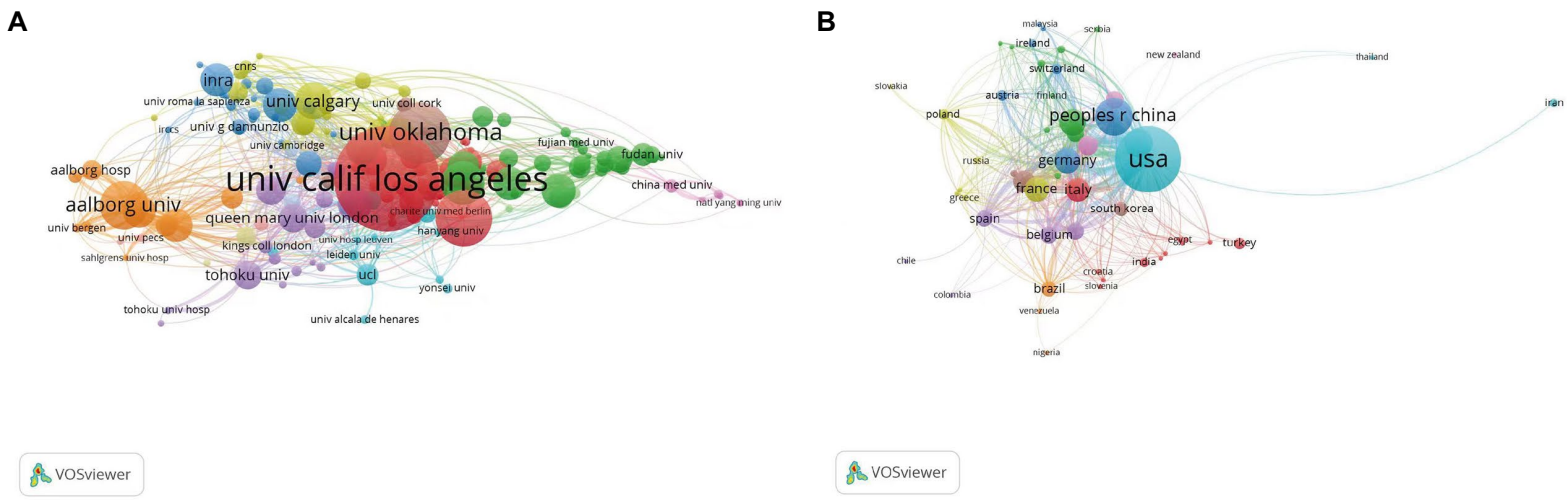
**(A)** The Network map of Institutions in visceral pain research (*T* ≥ 10). **(B)** The network map of countries in visceral pain research (*T* ≥ 5) Notes: The size of node reflects the institution’s or country’s published papers, the link indicate the collaborated relationship between institutions or countries, and the same color of node represent the same cluster.

In total, 4,035 institutions from 89 countries published research papers related to the field of visceral pain during the study period. The collaboration network between different institutions and countries was plotted using VOSviewer software optimization (Figures 4A,B), and we found that the United States, China, and the United Kingdom were the three largest nodes, representing the top three countries in terms of productivity in the field. The top 10 countries published a total of 4,512 articles (accounting for 89.40%), and the top five countries were the United States (1,716 articles), China (630 articles), the United Kingdom (376 articles), Japan (331 articles), and Germany (311 articles; Table 2). The top 10 institutions published a total of 741 (approximately accounting for 14.68%) visceral pain-related research papers, with the University of California (159 articles) publishing the most papers, followed by the University of Oklahoma (93 articles), the University of Pittsburgh (86 articles), Aalborg University (74 articles), and the University of Iowa (66 articles; Table 3). As shown in Figure 4A, a total of 194 institutions were divided into 13 clusters of different colors, and a total of 55 countries were divided into 9 clusters of different colors. This indicates that active and close cooperation is more common among top institutions and countries (Figures 4A,B), especially among institutions or countries in the same cluster.

**Table 3:** The top 10 productive institutions.

Rank	Institution	Country	*N*	Citations
1	University of California	United States	159	12,359
2	University of Oklahoma	United States	93	2,622
3	University of Pittsburgh	United States	86	3,560
4	Aalborg University	Denmark	74	2,542
5	University of Iowa	United States	66	4,275
6	University of Calgary	Canada	56	2,870
7	University of Adelaide	Australia	52	2,184
8	University of Manchester	United Kingdom	52	2,964
9	University of Texas	United States	52	2,984
10	Aalborg University Hospital	Denmark	51	1,455

*N*: The number of publications.

**Table 2:** The top 10 productive countries.

Rank	Country	Publications (*N*)	Citations
1	United States	1,716	74,784
2	China	630	8,684
3	United Kingdom	376	18,759
4	Japan	331	8,090
5	Germany	311	11,819
6	France	282	12,439
7	Italy	266	9,305
8	Canada	236	12,108
9	Australia	199	8,015
10	Denmark	165	5,893

### 3.4. Analysis of journals

**Figure 5:**
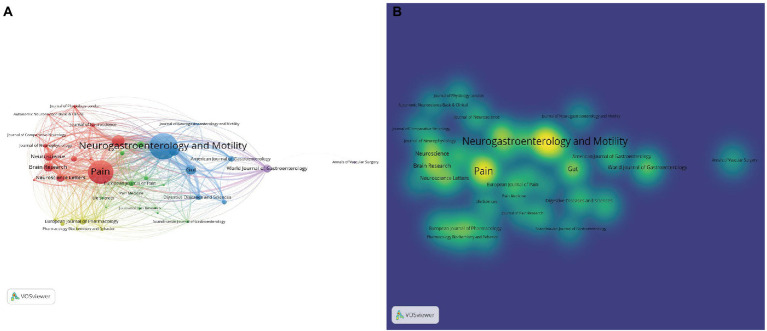
**(A)** The network map of journals in visceral pain research (*T* ≥ 20). The size of node reflects the journal’s published papers, the link indicates the collaborated relationship between journals, and the same color of node represent the same cluster. **(B)** The density map of journals in visceral pain research (*T* ≥ 20). The size of word, the size of round, and the opacity of yellow is positively related to the published papers.

In the analysis of journals, the visceral pain-related studies published between 1981 and 2021 were found to be published in 1,152 different journals. Collaboration between different journals was analyzed and a network map was constructed (Figure 5A), limiting the number of journal publications to greater than 20, and a total of 42 journals were included in the collaboration map. The same parameters were set to plot the density between journals (Figure 5B), again the same color represents the same cluster, the node size represents the number of papers, and the analysis of the results showed that there was a close cooperation between journals in the same cluster. Table 4 lists the top 10 journals in the field in terms of number of published articles, with a total of 1,268 published papers (accounting for 25.12%), and the highest impact factor (IF) journal was *Gastroenterology* (IF:33.88), whose JCR division was Q1. The top 5 journals in terms of number of published articles were *Neurogastroenterology and Motility* (276 articles), followed by *Pain* (252 articles), *American Journal of Physiology-Gastrointestinal and Liver Physiology* (119 articles), *Gastroenterology* (108 articles), and *Gut* (104 articles; Table 4). Thus, in terms of the number of articles published, *Neurogastroenterology and Motility* was the most popular journal in this field. Moreover, 70% of these 10 journals have a JCR partition of Q1 or Q2, which indicates that the quality and level of published journals for visceral pain-related research are relatively high, providing researchers in related fields with a reasonable and effective reference for journal selection.

**Table 4:** The top 10 productive journals.

Rank	Journal	Documents	IF	JCR
1	Neurogastroenterology and Motility	276	3.96	Q2/Q3
2	Pain	252	7.93	Q1
3	American Journal of Physiology-Gastrointestinal and Liver Physiology	119	4.87	Q1/Q2
4	Gastroenterology	108	33.88	Q1
5	Gut	104	31.79	Q1
6	Brain Research	92	3.61	Q3
7	Neuroscience	84	0.76	Q4
8	World journal of Gastroenterology	83	5.37	Q2
9	Neuroscience Letters	81	3.20	Q3
10	Plos one	69	3.75	Q2

### 3.5. Analysis of co-cited references and references burst

**Figure 6:**
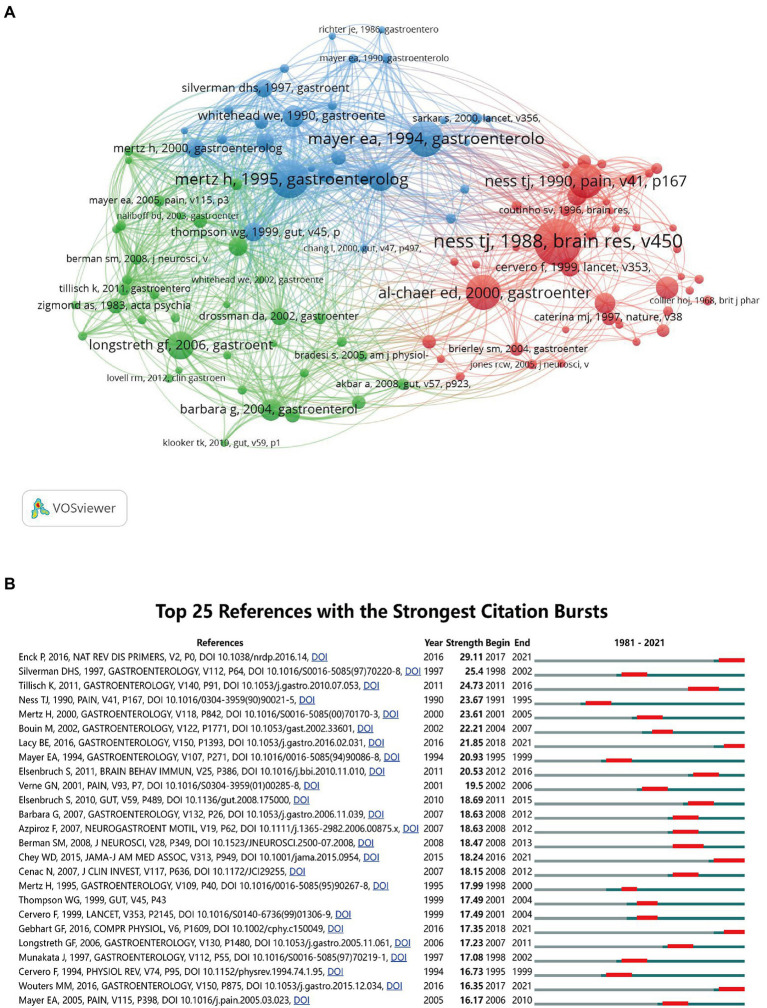
**(A)** The network map of co-cited references. Of the 111,351 references, 107 (classified into three clusters) had at least 50 times cited. **(B)** The top 25 references with the strongest citation bursts.

Cited literature is often considered a core component of bibliometric studies. There are 111,351 citations in the field, with a limited number of literature citations greater than 50. A collaborative network map of 107 literatures was optimized and plotted. The mapping analysis revealed some of the most important and influential references in research related to the field of visceral pain (Figure 6A). A total of three different clusters are depicted in Figure 6A, with the same color indicating the same cluster, representing references that are in similar research fields and are closely related to each other. The red cluster includes the most literature (42 articles), followed by the green cluster (36 articles), and the blue cluster (29 articles). Table 5 shows the top 10 cited papers (Ritchie, 1973; Zimmermann, 1983; Ness and Gebhart, 1988; Ness and Gebhart, 1990; Whitehead et al., 1990; Mayer and Gebhart, 1994; Mertz et al., 1995; Al-Chaer et al., 2000; Barbara et al., 2004; Longstreth et al., 2006). Five of them were cited more than 200 times, and the most cited article was a study published by Ness and Gebhart (1988) in *Brain Research* in 1988 on the mechanisms causing visceral stimulation, which showed that visceral stimulation caused by colorectal dilatation is an ethically acceptable methodology. The second most cited paper was by Mertz et al. (1995) who described the relationship between changes in rectal perception and IBS. The fourth most cited article was by Al-Chaer et al. (2000) who described that colonic stimulation in neonates can lead to chronic visceral hypersensitivity, providing a new animal model for IBS. The seventh most cited article was by Longstreth et al. (2006) on the diagnosis and treatment of functional bowel disease (FBD). The tenth was by Barbara et al. (2004) describing the mechanisms of abdominal pain in IBS. These papers were all related to IBS, indicating that early studies on IBS in the field of visceral pain were more numerous and focused mainly on mechanistic studies. The third (Mayer and Gebhart, 1994), sixth (Ritchie, 1973), and ninth (Whitehead et al., 1990) most cited articles were about the mechanisms related to nociceptive hypersensitivity, which is one of the important factors contributing to IBS. The fifth most cited article, published by Ness and Gebhart (1990), was a review on the mechanisms of visceral pain research. The eighth most cited article was published by Zimmermann (1983) on the ethical requirements for pain experiments. Most of this highly cited literature comprised of studies on the mechanisms of visceral pain, which provides a scientific and solid theoretical basis for future visceral pain-related research.

**Table 5:** The top 10 co-cited references on the research of visceral pain.

Rank	Title	Author	Year	DOI	Co-citation
1	Colorectal distension as a noxious visceral stimulus: physiologic and pharmacologic characterization of pseudaffective reflexes in the rat	Ness TJ and Gebhart GF	1988	10.1016/0006-8,993(88)91555-7	322
2	Altered rectal perception is a biological marker of patients with irritable bowel syndrome	Mertz H et al.	1995	10.1016/0016-5,085(95)90267-8	265
3	Basic and clinical aspects of visceral hyperalgesia	Mayer EA, and Gebhart GF	1994	10.1016/0016-5,085(94)90086-8	245
4	A new model of chronic visceral hypersensitivity in adult rats induced by colon irritation during postnatal development	Al-Chaer ED et al.	2000	10.1053/gast.2000.19576	241
5	Visceral pain: a review of experimental studies	Ness TJ and Gebhart GF	1990	10.1016/0304-3,959(90)90021-5	234
6	Pain from distension of the pelvic colon by inflating a balloon in the irritable colon syndrome	Ritchie J	1973	10.1136/gut.14.2.125	193
7	Functional bowel disorders	Longstreth GF et al	2006	10.1053/j.gastro.2005.11.061	190
8	Ethical guidelines for investigations of experimental pain in conscious animals	Zimmermann M	1983	10.1016/0304-3,959(83)90201-4	154
9	Tolerance for rectosigmoid distention in irritable bowel syndrome	Whitehead, WE et al.	1990	10.1016/0016-5,085(90)90332-u	152
10	Activated mast cells in proximity to colonic nerves correlate with abdominal pain in irritable bowel syndrome	Barbara G et al.	2004	10.1053/j.gastro.2003.11.055	149

Burst citations refers to literature that has been frequently cited over time and can be used to indicate the evolution of a field of knowledge (Synnestvedt et al., 2005). Figure 6B lists the top 25 pieces of literature in terms of citation burst intensity, with the highest citation intensity (29.11) for IBS-related studies published by Enck et al. (2016). This article summarized the mechanisms of action of IBS, including the potential roles of gut microbiota groups, the brain-gut axis, genetics, and inflammation, and discussed current diagnostic approaches and treatment options. It signals a potential continued increase in future IBS-related research in visceral pain. Silverman et al. (1997) published an article with the second highest citation intensity, which focused on the fact that visceral pain may affect anterior cingulate cortex (ACC) activity in the brain. Tillisch et al. (2011) published an article with the third highest citation intensity, and this study showed that IBS causes activation in relevant areas of the brain (thalamus, insula, anterior cingulate cortex, and midbrain). These analyses suggest that these studies are also valuable guides and references for future studies related to visceral pain.

### 3.6. Analysis of keywords and hotspots

**Figure 7:**
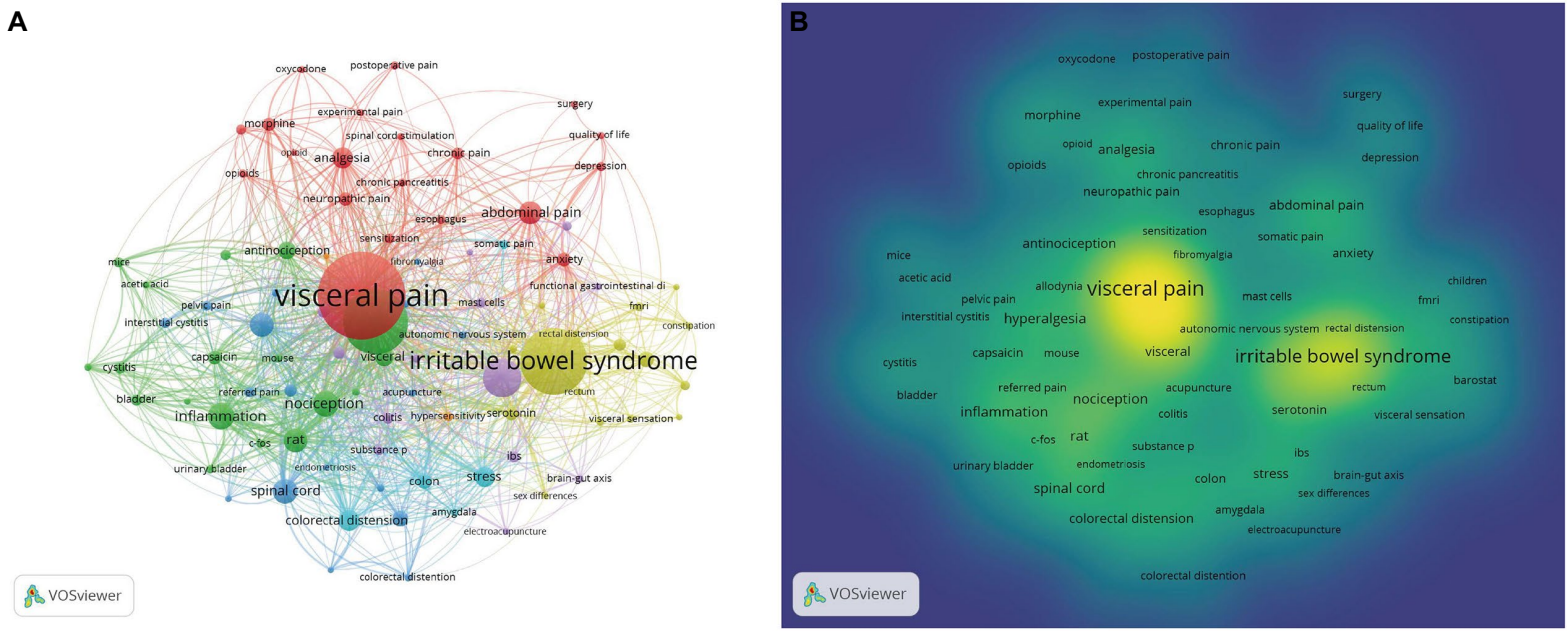
**(A)** The co-occurrence map of keywords in visceral pain research (*T* ≥ 20) Notes: The size of node reflects the keyword’s co-occurrence frequencies, the link indicates the co-occurrence relationship between keywords, and the same color of node represent the same cluster. **(B)** The density map of keywords in visceral pain research (*T* ≥ 10). The size of word, the size of round, and the opacity of yellow is positively related to the co-occurrence frequencies.

The co-occurrence network mapping was performed based on keywords, with a total of 8,367 keywords included, and the threshold for the number of occurrences was set to greater than 20. Finally, only 91 keywords were included in the network mapping, including seven different clusters (Figure 7A). In the figure, the size of the node represents the frequency of the keyword; the larger the node, the more frequently the keyword appears. The same color represents the same cluster, and the same clusters are closely related to each other and point in similar directions. The first cluster is in red and describes a total of 20 keywords related to different types of visceral pain, including abdominal pain, analgesia, cancer pain, and chronic pain. The second cluster is in green, with a total of 18 keywords mainly related to *in vivo* experimental research of pain, including mice, analgesia, and rats. The third cluster is blue, and mainly contains a total of 17 keywords related to hyperalgesia, including pelvic pain, endometriosis, fibromyalgia, and abnormal pain. The fourth cluster is yellow, which mainly summarizes a total of 16 keywords of IBS-related influencing factors, including brain, children, constipation, rectal dilatation, gender differences, and visceral sensitization. The fifth cluster is purple, which mainly describes a total of 13 keywords related to intestinal diseases, including colitis, brain-gut axis, IBD, and functional bowel disease. The sixth cluster is blue-green and shows a total of 5 keywords related to stress, including colorectal dilatation, amygdala, stress, and colon. The seventh cluster is orange and summarizes a total of two keywords related to pancreatitis: pancreatitis and hypersensitivity. The keyword co-occurrence network diagram was further used to draw a correlation density map (Figure 7B) and the same high frequency co-occurrence keywords could be found. Table 6 summarizes the top 25 keywords that appeared in studies related to visceral pain, with the top 5 keywords being visceral pain (631 times), IBS (441 times), pain (423 times), visceral hypersensitivity (214 times), and inflammation (133 times).

**Table 6:** The top 25 keywords related to visceral pain.

Rank	Keyword	Frequency
1	Visceral pain	631
2	Irritable bowel syndrome	441
3	Pain	423
4	Visceral hypersensitivity	214
5	Inflammation	133
6	Nociception	125
7	Spinal cord	120
8	Rat	114
9	Hyperalgesia	112
10	Abdominal pain	111
11	Colorectal distension	98
12	Analgesia	98
13	Stress	95
14	Visceral	80
15	Antinociception	75
16	Visceral hyperalgesia	66
17	Visceral sensitivity	65
18	Colon	63
19	Neuropathic pain	58
20	Capsaicin	57
21	Anxiety	57
22	Serotonin	51
23	Morphine	51
24	Visceral nociception	49
25	Chronic pain	47

**Figure 8:**
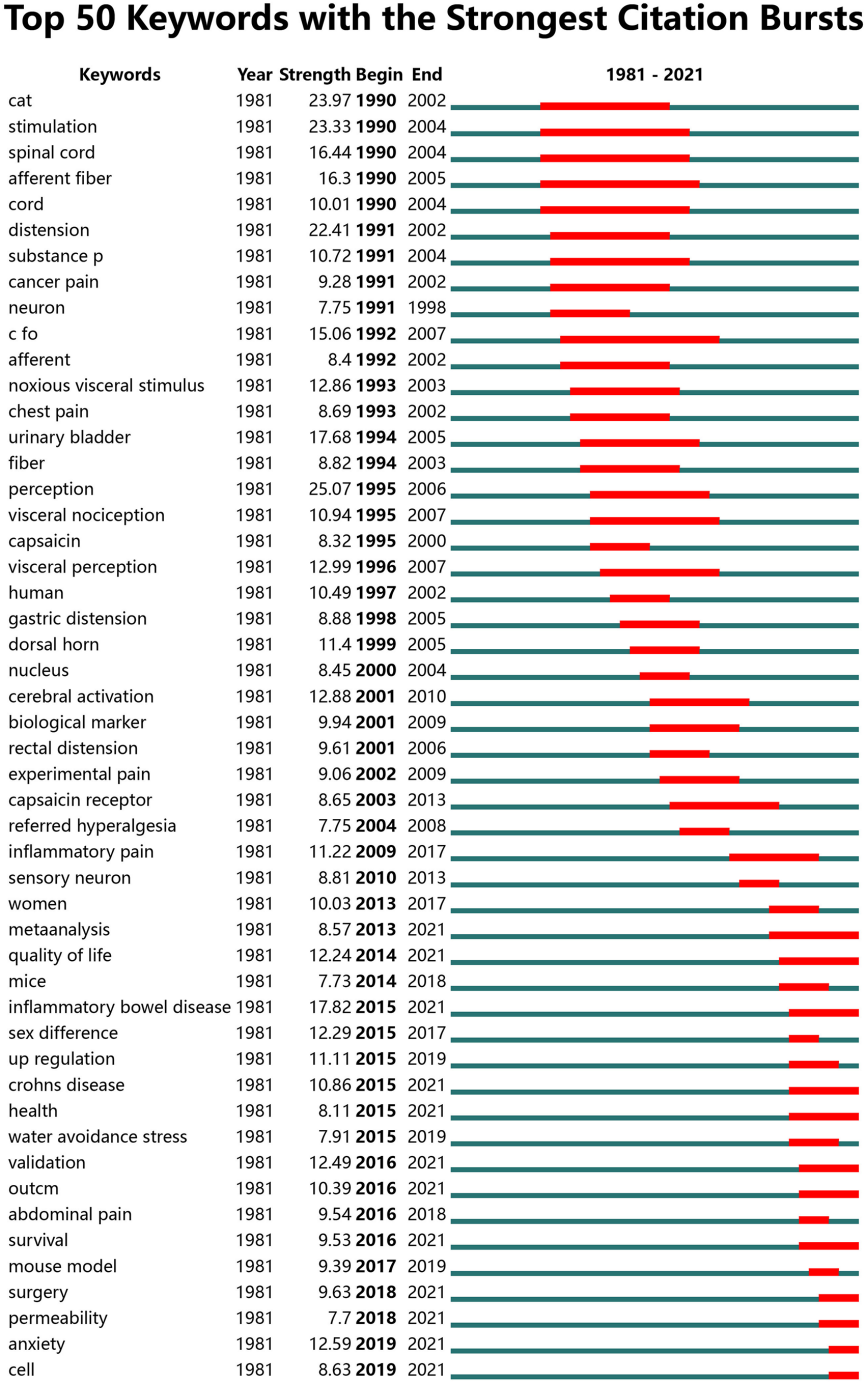
The top 50 keywords with strongest citation bursts.

In addition, we also used CiteSpace to draw the evolution map of emerging high-intensity keywords, describing the current research hotspots and trends in this field. Figure 8 shows the top 50 keywords by emergence strength, and the red line indicates the duration of the emergent keywords. Among them, the keyword “perception” had the highest intensity (Strength:25.07), and the second to fifth ranked keywords were cat (Strength:23.97), irritation (Strength:23.33), dilation (Strength:22.41), and inflammatory bowel disease (Strength:17.82), respectively. In addition, the emergent keywords that lasted until 2021 were meta-analysis (2013–2021), quality of life (2014–2021), inflammatory bowel disease (2015–2021), Crohn’s disease (2015–2021), and anxiety (2019–2021); of these, the keyword “inflammatory bowel disease” had the strongest emergent intensity of 17.82 compared to other keywords that lasted until 2021, indicating that inflammatory bowel disease is a recent research hotspot in the field of visceral pain. The results of the emergent keyword map indicate that in recent years there has been an increasing interest in the study of the relationship between IBD (Strength:17.82), anxiety (Strength:12.59), quality of life (Strength:12.24), and visceral pain. Comprehensive keyword and research hotspot analysis reveals that in the field of visceral pain, inflammation, visceral hypersensitivity, IBD, IBS, anxiety, and quality of life may be the future research hotspots. In order to further clarify the trend of these hot spots over time, we did the analysis of each keyword in the time period (Supplementary Figures S1, S2). This provides scientific and research guidance and future research directions for scholars in the fields related to pain, digestive diseases, and neuroscience diseases.

## 4. Discussion

Visceral pain is a complex and heterogeneous disorder, which is considered more prominent than somatic pain and is accompanied by emotional and mood disorders (Schmidt et al., 2022). As the burden of visceral pain increases, an increasing number of researchers are studying visceral pain (Pusceddu and Gareau, 2018; Shaballout et al., 2021; Ceuleers et al., 2022; Jain et al., 2022; Long et al., 2022; Morreale et al., 2022; Zhang et al., 2022). Thus, a systematic analysis of topics in this field using bibliometric methods has been used in this study to gain a clearer understanding of the current state of research and future research trends in the field. In this study, we searched the relevant literature from the WOS core database and investigated the developments and hotspots in the field of visceral pain through bibliometrics.

Visceral pain research has been conducted for more than 20 years to date, with visceral pain studies gradually emerging since 1988. Publications by Ness and Gebhart (1988) showed that colorectal distension-induced visceral stimulation is an ethically acceptable methodology in rats. Subsequently, they conducted experiments on people and found that pain was correlated with the amount of pressure administered and persisted after the pressure was withdrawn (Ness et al., 1990). Since then, there has been a significant increase in the study of visceral pain. As shown in Figure 2, the cumulative number of publications has rapidly and steadily increased since 1991 which suggested that visceral pain research attracted great research interest. In the past decade, the most cited article is by Gerald F. Gebhart, whose influence on research in the field of visceral pain has been significant by proposing a method for simulating visceral pain and assessing pain caused by colon dilation through visceral motor responses (Ness and Gebhart, 1988; Christianson and Gebhart, 2007). It lays a foundation for future research on visceral pain. Based on the distribution of countries, we found that the United States dominates the output of the field in terms of volume. Although the United States produced the largest number of publications related to visceral pain, China and the United Kingdom also contributed a significant number of publications in this area. Research institutions from the United States (University of California, University of Oklahoma, and University of Pittsburgh) dominate visceral pain research. The journals analyzed that found the most articles on visceral pain were in *Neurogastroenterology and Motility*, followed by *Pain* and the *American Journal of Physiology - Gastrointestinal and Liver Physiology*. This shows that the study of pain in digestive disorders is a current hot topic. Also, 70% of the articles in the top 10 journals had a JCR division of Q1 or Q2, indicating that most of the studies were from high-impact journals, suggesting that visceral pain-related research is of high value in the global research field. All these journals are closely linked (Figure 5). Overall, the authors, institutions, countries, and journals in the field of visceral pain research are mostly dominated by Western countries, such as USA, UK, Germany, Denmark. In contrast, most of the eastern countries are in China, mainly Prof. Guang-Yin Xu from Soochow University, who studies the neuromolecular mechanisms of functional visceral pain, including IBS (Li et al., 2022), maternal–infant segregation (Du et al., 2019) and brain-gut axis (Xu et al., 2022), etc. Therefore, it is recommended to enhance global collaboration, communication, and cooperation among research teams. By far the most highly cited study was that by Ness and Gebhart (1988) published in Brain Research in 1988 on the mechanisms causing visceral stimulation, followed by Mertz et al. (1995) describing the relationship between changes in rectal perception and IBS, and Mayer and Gebhart (1994) describing the mechanisms involved in nociceptive hypersensitivity. This high number of citations in the literature suggests that the most used model in the field of visceral pain research is probably colorectal dilatation, which may lead to changes in bowel perception, forming nociceptive sensitization that eventually evolves into IBS. Indeed, IBS is known to be one of the commonest types of visceral pain (Abuelazm et al., 2022). On this basis, a network map was further constructed for visualization to generate key clusters of co-cited literature and visualization of the timeline of co-cited literature, which were divided into three major clusters, each closely related to the other. Red clustering is dominated by the study published by Ness and Gebhart (1988) in *Brain Research* on the mechanisms involved in causing visceral stimulation. Blue clustering is dominated by the publication of mechanisms related to nociceptive hypersensitivity by Mertz et al.(Mertz et al., 1995) in *Gastroenterology*. The green clustering is based on the study of functional enteropathy published by Longstreth et al. (2006) in *Gastroenterology*. It can be seen that the visceral stimulation mechanism studied by Ness and Gebhart (1988) was the first to be cited. Among the more cited literature in recent years includes a summary of the mechanisms of functional bowel disease by Enck et al. (2016). Mearin et al. (2016) reclassified and updated the clinical evaluation and treatment of functional bowel disease. Chey et al. (2015) reported the etiology, symptoms, and improvement of IBS. Gebhart and Bielefeldt (2016) described the functional anatomy and central processing mechanisms of visceral pain. Wouters et al. (2016) found that histamine receptor H1 (HRH1)-mediated transient reporter potential channel V1 (TRPV1) sensitization was associated with IBS, and that application of an antagonist of HRH1 reduced visceral hypersensitivity and abdominal pain; now in early clinical trials, this may become a new treatment for IBS. However, studies have shown that non-pharmacological treatments for IBS pain include a low-FODMAP (Fermentable oligosaccharides, disaccharides, monosaccharides, and polyols) diet, probiotics, and psychological interventions, especially hypnotherapy. Tricyclic drugs remain the best medical treatment option, and for severe pain, consider the concept of combining gut-cranial neuromodulators and psychotherapy in a multidisciplinary context (Paine, 2021). In general, the new therapeutic targets for visceral pain are also the research hotspots of researchers in recent years. These recent citations range from basic mechanistic research to clinical treatments, with the latter indicating that visceral pain research has begun to gradually refine and move closer to the clinic.

The evolution of a research field can be understood by a change in keywords, through which we can gain an understanding of the topic of the article, and reveal the future emerging hot spots and trends through keyword analysis. Keyword co-occurrence analysis showed that the high frequency keywords included visceral pain, IBS, pain, visceral hypersensitivity, and inflammation. The keyword co-occurrence network mapping revealed that these high-frequency keywords were located in the middle of each cluster and are important core terms, reflecting the area of interest in the research field. Most of these studies have focused on exploring the mechanisms that cause visceral pain. Through keyword burst analysis, the keywords of the study change over time, from which we can understand the research hotspots and development trends of each period. From 1990 to 2000, research mainly focused on the potential mechanism of visceral pain, especially for IBS. For example, IBS patients were found to have rectal distension sensitivity and hypersensitivity (Naliboff et al., 1997). IBS with rectal distention is associated with ACC activation in the brain (Silverman et al., 1997). In addition, the lack of specific biomarkers for IBS is proposed, and IBS is suggested to be classified according to bowel habits, which provides guidance for further improvement of the diagnosis of irritable bowel syndrome (Schmulson et al., 2000). From 2001 to 2011, it was gradually found that visceral pain may involve brain mechanisms and some neuronal effects. A 2005 study found that neurons in the ventral medulla caudalis encode nociceptive information from visceral and cutaneous structures, and that most of these neurons are stimulated by both bladder and colorectal distention (Robbins et al., 2005). In 2006, Robbins et al. (2006) found that the response neurons induced by bladder distention were located in the ventral basal thalamus group. In addition, a 2011 study found that various experimental nociceptive conditioning stimuli to the skin, muscle, or internal organs produced hyperalgesia and resulted in secondary changes in the brain (Woolf, 2011). From 2012 to 2021, visceral pain research covered many aspects, including gender differences, the impact of quality of life and the generation of negative emotions. For example, Jiang et al. (2013) found that men and women produce pain through different mechanisms. Pain in women may be mainly associated with enhanced perception and ease of sensory signals, whereas in men it is mainly associated with autonomic hyperresponsiveness, and Yaklai et al. (2021) showed that IBS can significantly reduce the quality of life of patients. Huang et al. (2020) reported that anxiety and chronic visceral pain often co-exist in clinical studies. A 2022 review suggests that brain gut axis two-way loop is connected to the gastrointestinal tract and central nervous system, the epigenome regulation in affect brain gut axis of development in the future of clinical disease diagnosis and treatment of intervention plays an important role, and the future of the next generation of group learning method and the progress of the bioinformatics could be an important means of the mechanism of visceral pain (Higgins et al., 2022). In addition, through the time trend of keywords, it is found that the research direction in the field of visceral pain has gradually shifted from basic disease mechanism research to clinical treatment research (next-generation multi-omics methods and bioinformatics). This suggests that visceral pain research is no longer limited to the study of internal body mechanisms, and more attention has been paid to its psychological impact on people in recent years, such as anxiety. In summary, it is recommended that researchers in the field of visceral pain pay attention to scientific research hotspots and that research institutions strengthen communication and cooperation to promote the academic development of this field.

### 4.1. Strengths and limitations

This study is the first visual analysis of the field of visceral pain research based on literature published from 1981 to 2021, analyzed from different perspectives, including global trends, authors, authorities, countries, published journals, cited references, and keywords. The current state of research in the field of visceral pain has been explored through several aspects. However, this study also has limitations. For example, the data analyzed were limited to the WOS core database, which is not comprehensive. In addition, all the data were obtained through analysis tools based on bibliometric software, which may lead to biases discussed in other bibliometric studies.

## 5. Conclusion

In general, in the study of visceral pain, the academic community mainly focuses on some clinical diseases (IBD, IBS, anxiety, etc.). Bibliometric analysis helps scholars to understand academic cooperation, research trends and hot issues from numerous literatures. In this study, we summarized the contributing countries, affiliations, authors, journals, and citation data for visceral pain research. Research groups from the United States are important contributors to the development of this field. In order to obtain more high-quality research results, international collaboration should be strengthened. Current publications are focused on molecular, biological, and clinical features. Discovering specific molecular mechanisms between visceral pain and anxiety in the future, as well as molecular targets for the treatment of clinical disorders related to visceral pain, may become major research hotspots and possible directions for future development.

This study is the first study to examine the literature related to visceral pain through bibliometric and knowledge mapping systems. Our study provides objective and original insights into the field of visceral pain research. The results of this study will provide valuable references and directions for future research.

## Data availability statement

The raw data supporting the conclusions of this article will be made available by the authors, without undue reservation.

## Author contributions

LG: draft preparation and data analysis. YL and BW: data analysis and drawing. AC: data analysis. WT and CL: supervision, editing, and writing the manuscript. All authors contributed to the article and approved the submitted version.

## Funding

This research was supported by Fujian Science and Technology Innovation Joint Fund (2018Y9069) and Fujian Provincial Financial Special Fund 2019B028.

## Conflict of interest

The authors declare that the research was conducted in the absence of any commercial or financial relationships that could be construed as a potential conflict of interest.

## Publisher’s note

All claims expressed in this article are solely those of the authors and do not necessarily represent those of their affiliated organizations, or those of the publisher, the editors and the reviewers. Any product that may be evaluated in this article, or claim that may be made by its manufacturer, is not guaranteed or endorsed by the publisher.

## Supplementary material

SUPPLEMENTARY FIGURE S1 The overlay visual network of keywords (The circle size represents the frequency of keywords, and different colors represent different time periods).

SUPPLEMENTARY FIGURE S2 (A) The overlay visual network of keywords (core: inflammation). (B) The overlay visual network of keywords (core: visceral hypersensitivity). (C) The overlay visual network of keywords (core: IBD). (D) The overlay visual network of keywords (core: IBS). (E) The overlay visual network of keywords (core: anxiety). (F) The overlay visual network of keywords (core: quality of life).

SUPPLEMENTARY FIGURE S3 The flowchart of bibliometric analysis.
