# Supplementary figures and images for: Research hotspots and trends in visceral pain research: A global comprehensive bibliometric analysis
Source: Front Mol Neurosci. 2023 Jan 4;15:1022463. doi: 10.3389/fnmol.2022.1022463 (PMC9848657; doi:10.3389/fnmol.2022.1022463)

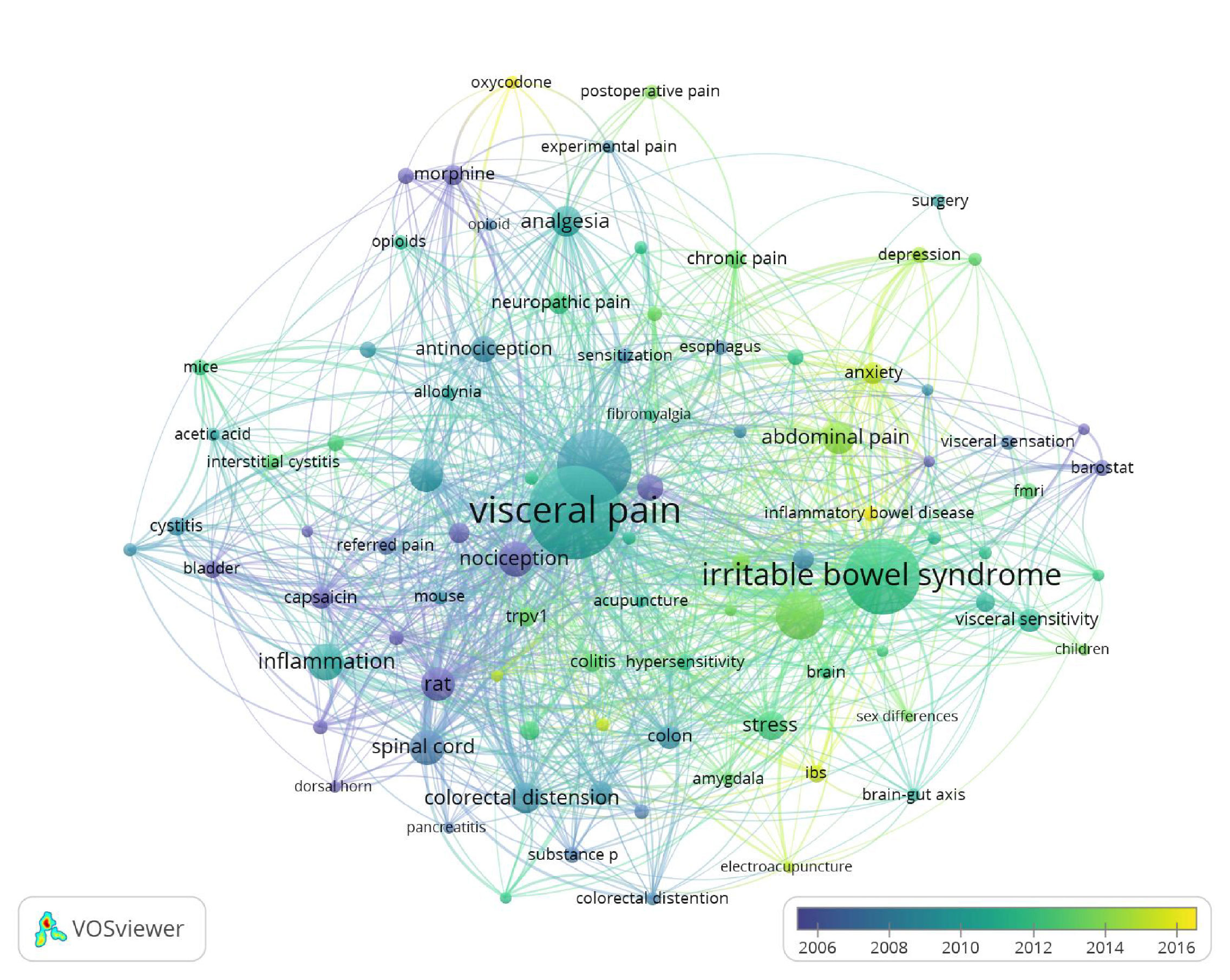

Supplement: SUPPLEMENTARY FIGURE S1 — The overlay visual network of keywords (The circle size represents the frequency of keywords, and different colors represent different time periods). [file Image_1.JPEG]

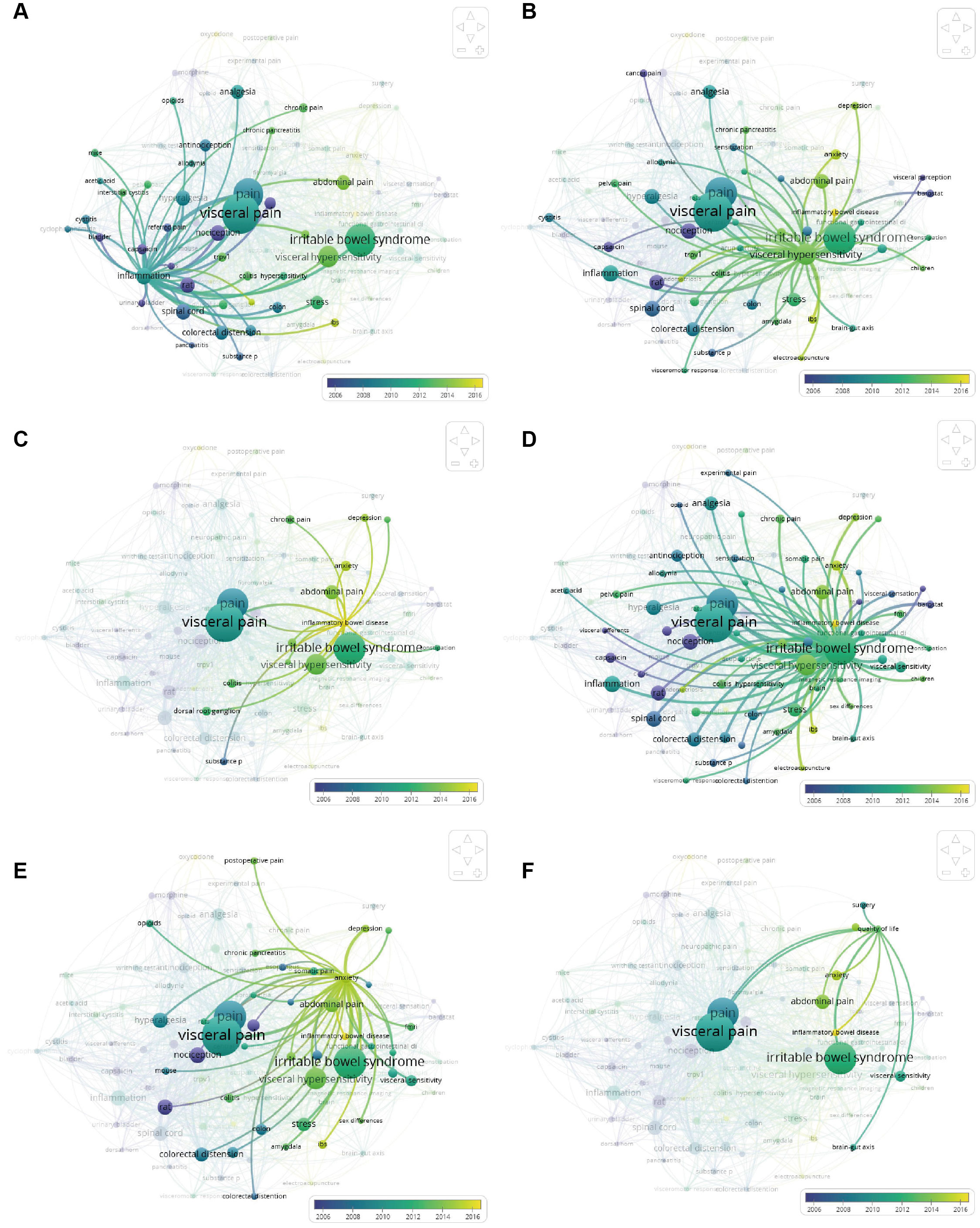

Supplement: SUPPLEMENTARY FIGURE S2 — (A) The overlay visual network of keywords (core: inflammation). (B) The overlay visual network of keywords (core: visceral hypersensitivity). (C) The overlay visual network of keywords (core: IBD). (D) The overlay visual network of keywords (core: IBS). (E) The overlay visual network of keywords (core: anxiety). (F) The overlay visual network of keywords (core: quality of life). [file Image_2.JPEG]

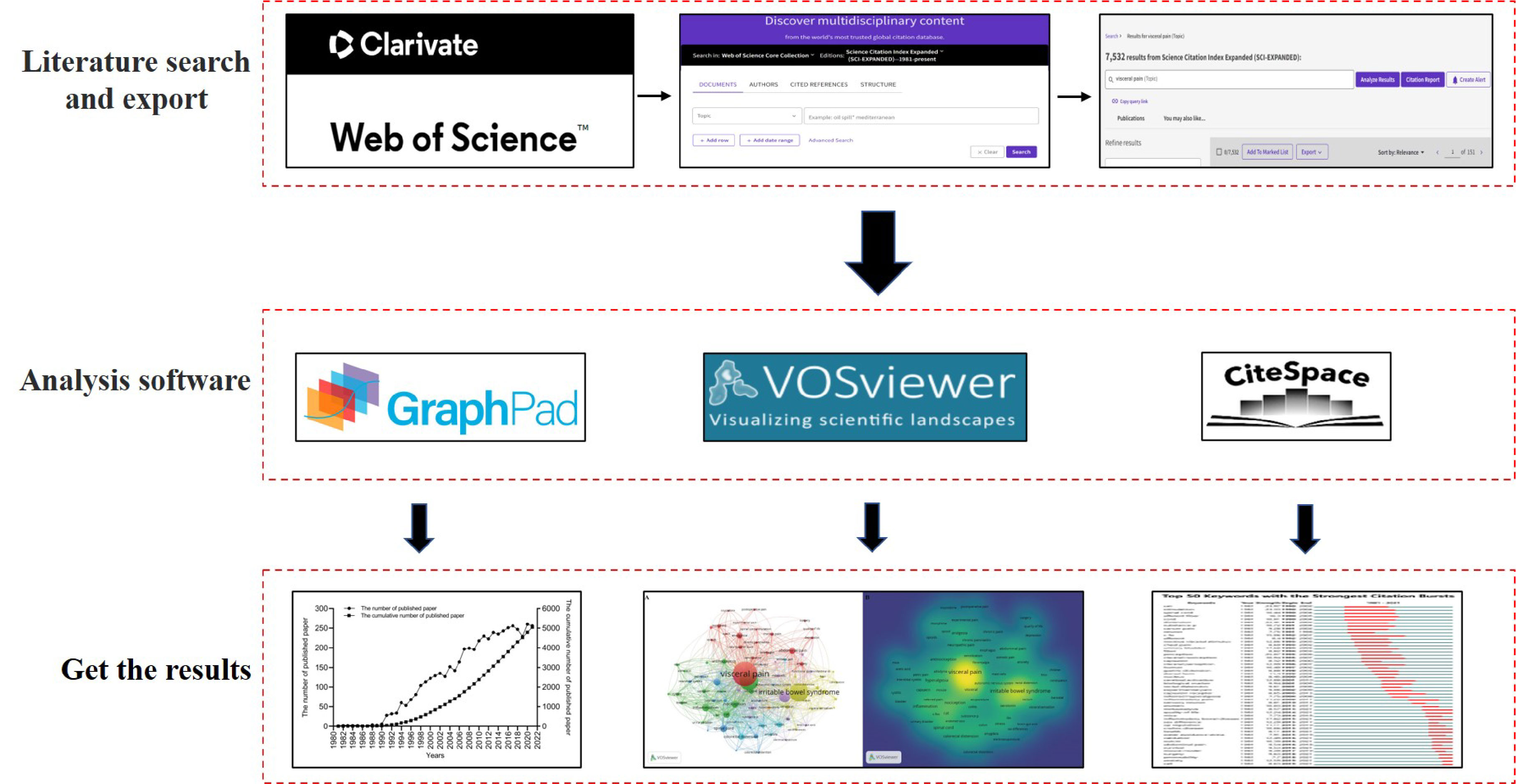

Supplement: SUPPLEMENTARY FIGURE S3 — The flowchart of bibliometric analysis. [file Image_3.JPEG]
